# PSOSP uncovers pervasive SOS‐independent prophages with distinct genomic and host traits in bacterial genomes

**DOI:** 10.1002/imt2.70073

**Published:** 2025-08-18

**Authors:** Yali Hao, Mujie Zhang, Xinjuan Lei, Chengrui Zhu, Taoliang Zhang, Yanping Zheng, Xiang Xiao, Huahua Jian

**Affiliations:** ^1^ State Key Laboratory of Microbial Metabolism, Joint International Research Laboratory of Metabolic & Development Sciences, School of Life Sciences and Biotechnology Shanghai Jiao Tong University Shanghai China; ^2^ Yazhou Bay Institute of Deepsea Science and Technology, Hainan Research Institute Shanghai Jiao Tong University Sanya China; ^3^ Center for Precision Medicine The First Affiliated Hospital of Xiamen University, School of Medicine, Xiamen University Xiamen China

## Abstract

Prophages are ubiquitously present in bacterial genomes, significantly influencing host physiological and ecological functions. We present Prophage SOS‐dependency Predictor (PSOSP), a novel bioinformatics tool that predicts prophages induction modes by analyzing the Heterology Index (*HI*) of LexA protein binding to target DNA, classifying prophages into SOS‐dependent prophages (SdPs) and SOS‐independent prophages (SiPs). PSOSP was experimentally validated to accurately distinguish SdPs from SiPs in a test set, achieving 100% sensitivity and specificity. Applying PSOSP to 49,333 complete bacterial genomes, we inferred prophage induction modes in 15,493 bacterial genomes and identified 11,806 SiPs. These SiPs are widely distributed across 145 bacterial genera and exhibit distinct genomic features compared to SdPs. Correspondingly, the hosts of these two prophage types are hypothesized to differ in their physiological characteristics. These findings, powered by PSOSP, provide not only novel insights into the diverse induction mechanisms but also a critical methodology for future phage–host interaction studies.

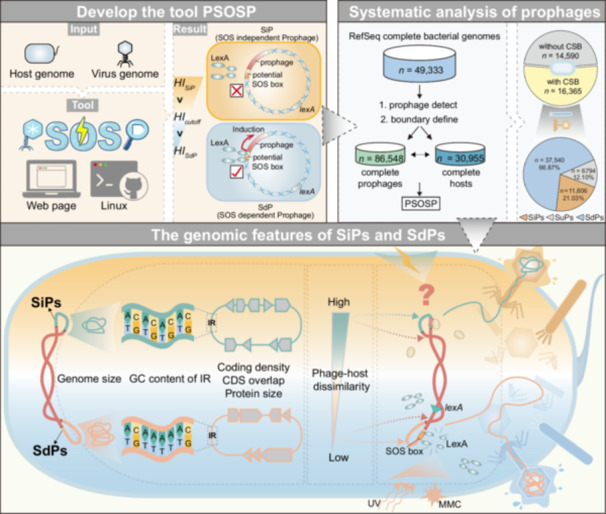

To the Editor,

Prophages, the dormant form of temperate phages during the lysogenic cycle, are widely present in bacterial genomes in either integrated or nonintegrated forms [1, 2]. These prophages can be induced into an active state in response to environmental changes, leading temperate phages to enter the lytic cycle and thereby generating significant ecological impacts. This process is referred to the lysogenic‐lytic switch [3]. The classical lysogenic‐lytic switch relies on the bacterial SOS pathway (Figure 1A), which is activated in response to DNA‐damaging agents such as ultraviolet (UV) radiation and mitomycin C (MMC), initiating a process for DNA repair [4]. The LexA protein is a key regulator of the SOS pathway, controlling the transcriptional activation of dozens of genes, including *recA* and *recN* [5]. The promoter regions of SOS pathway genes regulated by LexA often contain conserved sequences for LexA binding (in *Escherichia coli*, 5'‐TACTG(TA)_5_CAGTA‐3'), known as the SOS box [6]. Therefore, the core components of the SOS pathway, namely LexA and RecA, are considered critical regulators of the prophage lysogenic‐lytic switch [7, 8, 9]. Based on this mechanism, traditional prophage induction experiments typically employ DNA‐damaging agents (such as UV or MMC) to activate the SOS pathway, thereby inducing prophages into the lytic cycle. The outcomes of these experiments are often used to evaluate the lysogeny capacity of bacteria [10]. However, in recent years, SOS pathway‐independent induction of prophages has been reported [11, 12]. Furthermore, induction experiments using MMC on environmental microorganisms have shown highly variable induction rates, with widespread insensitivity [10]. Nevertheless, due to the lack of specialized tools for determining prophage induction modes, the distribution proportions and genomic characteristics of SOS‐independent prophages (SiPs) remain largely unexplored. Consequently, developing universal tools to characterize phage induction modes is essential for systematically elucidating the biological traits of temperate phages.

**FIGURE 1:**
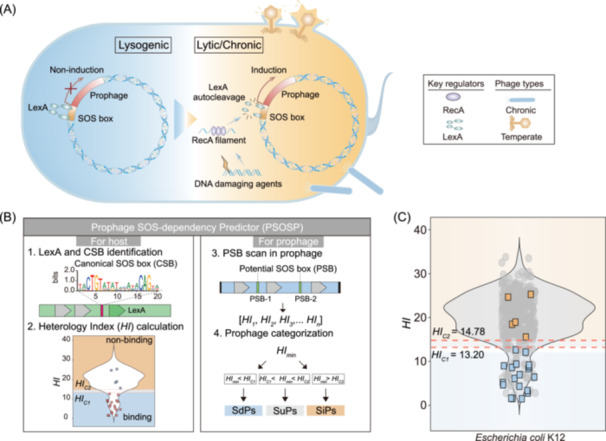
The principle of Prophage SOS‐dependency Predictor (PSOSP) and its workflow schematic diagram. (A) Schematic diagram of the currently known lysogenic‐lytic switch mechanism in temperate phages. Temperate phages integrate into the bacterial host genome as prophages. Under normal conditions, the LexA protein binds to the SOS box within the prophage, repressing the expression of phage‐related genes and maintaining the lysogenic state. Upon external stimuli (such as exposure to DNA‐damaging agents), the RecA protein is activated, leading the self‐cleavage of LexA and its dissociation from the SOS box. This relieves the prophage repression, triggering the temperate phage to enter the lytic cycle and thereby facilitating its proliferation. (B) Schematic diagram of the PSOSP workflow. The workflow consists of four main steps: (1) scanning the host genome to identify LexA protein and canonical SOS boxes (CSBs) located upstream of the *lexA* gene; (2) identifying potential SOS boxes (PSBs) across bacterial genomes, calculating the Heterology Index (*HI*) for each PSB and establishing classification thresholds (*HI*_C1_ and *HI*_C2_) via Mean Shift clustering results; (3) scanning PSBs within prophage promoter regions and determining of the minimum *HI* (*HI*_min_); and (4) evaluating the ability of LexA binding to prophage promoter regions by comparing *HI*_min_ with thresholds (*HI*_C1_ and *HI*_C2_), and subsequently classifying prophages as SOS‐dependent prophages (SdPs), SOS‐independent prophages (SiPs), or SOS‐uncertain prophages (SuPs). Red dashed lines indicate the upper threshold of the low‐*HI* category (*HI*_C1_) and the lower threshold of the high‐*HI* category (*HI*_C2_). (C) Distribution of *HI* values for all PSBs in *E. coli* K12. Experimentally validated PSBs that are capable and incapable of LexA binding are represented by blue and orange squares, respectively.

Previous studies have indicated that in *E. coli*, the binding likelihood of a DNA sequence to the LexA protein can be evaluated based on its degree of divergence from the consensus SOS box, quantified as Heterology Index (*HI*). This metric further allows for inference regarding its regulation by LexA and the SOS response [13]. Additionally, although the gene composition of the LexA regulon varies among different bacterial taxa, the sequence of the SOS box remains relatively conserved across multiple bacterial taxa [14, 15, 16]. Inspired by this evidence, we propose that in bacteria harboring LexA and canonical SOS boxes (CSBs), the presence of potential SOS boxes (PSBs) in prophage promoter regions can be used to evaluate whether prophage induction is dependent on the SOS response by calculating *HI* (Figure 1B). Accordingly, we developed a novel bioinformatics tool, termed the Prophage SOS‐dependency Predictor (PSOSP) (https://vee-lab.sjtu.edu.cn/PSOSP/), to facilitate this analysis. Using PSOSP, we conducted a comprehensive survey of 49,333 complete bacterial genomes and successfully predicted the induction modes of prophages in 15,493 genomes, identifying 11,806 SiPs distributed across 145 bacterial genera. These SiPs exhibit distinct genomic features compared to SOS‐dependent prophages (SdPs). Our findings refine the conventional understanding of temperate phage induction mechanisms and provide novel tools and insights for exploring the lysogenic‐lytic switch of phages.

## *HI* reliably predicts LexA binding potential

To validate the reliability of *HI* in predicting LexA binding capability, we first analyzed the *HI* of all PSBs in *E. coli* strain K12 substrain. MG1655 (NCBI accession: GCF_002843685.1) [17], revealing a bimodal distribution comprising sequences with high *HI* (peak at 22.32) and low *HI* (peak at 7.34), which likely represents DNA fragments capable of binding to LexA (Figure 1C). To determine the *HI* threshold, we conducted statistical analyses using seven methods, which demonstrated that the Mean Shift method provided the optimal discrimination (Figure S1, Tables S1–2, and Supplemental Note 1). Comparison with DNA‐protein binding experimental results, which were previously reported by Lewis et al. in *E. coli* [17], confirmed an accuracy of 100% (*n* = 24), with a 95% confidence interval of 85.8% to 100% (Figure 1C and Table S3).

To extend this validation beyond model organisms, we further assessed *HI* reliability using the prophage SW1 from the deep‐sea bacterium *Shewanella piezotolerans* WP3 for analysis. SW1 is a filamentous phage isolated from deep‐sea sediments; its gene expression and virion production are induced by low temperature and high pressure [18]. Genomic analysis of SW1 identified three PSBs, all of which exhibited *HI* values higher than the threshold observed in its host *S. piezotolerans* WP3 (Figure S2A,B), suggesting that SW1 DNA lacks the ability to bind LexA. This conclusion was confirmed by electrophoretic mobility shift assay (EMSA) experiments using purified LexA protein and the three PSBs (Figure S2C). Site‐directed mutagenesis experiments on these PSBs demonstrated that modifying them to achieve *HI* values below the threshold conferred LexA binding capability (Figure S2B,D,E). Collectively, these findings indicate that *HI* can indeed serve as a reliable indicator of LexA binding potential.

## PSOSP: Determining the regulatory mode of prophages based on *HI*

To evaluate the accuracy of PSOSP in classifying prophages, we collected prophages with existing experimental data, including ten prophages (8.5–62.8 kb) spanning eight viral taxonomic families, thus representing diverse prophage groups (Table S4). PSOSP accurately identified eight SdPs and two SiPs, fully aligning with published data (Table S4), achieving 100% sensitivity and 100% specificity in the test set (*n* = 10) (Figure S3).

Furthermore, analysis of SW1, classified by PSOSP as a SiP, revealed that while the classical SOS inducers UV and MMC significantly upregulated the transcription of *recA* (*p* = 0.030 and 0.008 for UV and MMC treatment, respectively) and *lexA* (*p* = 0.014 and 0.004 for UV and MMC treatment, respectively) in its host *S. piezotolerans* WP3 (Fold change > 4), they failed to activate key genes expression in SW1 (Fold change ≤ 1). This confirmed that SW1 was indeed a SiP, a conclusion further supported by experiments involving *lexA* mutants (Figure S2F). To further evaluate PSOSP's performance in polylysogens (bacteria containing multiple prophages in their genomes), we analyzed the deep‐sea bacterium *S. psychrophile* WP2, which harbors three integrated prophages: SP1 (42.6 kb), SP2 (38.8 kb), and SP3 (8.1 kb). SP1 and SP2 exhibit typical genomic organizations of tailed phage, with SP2 identified as a transposable phage, while SP3 encodes the Zot protein and is speculated to be a filamentous phage. PSOSP classified two prophages, SP1 and SP3, as SiPs, while SP2 was categorized as a SdP (Figure S4A,B). MMC induction and Virion Induction Profiling Sequencing (VIP‐Seq) quantitative analysis confirmed that SP2 was significantly induced by MMC (*p* = 0.007), whereas SP1 (*p* = 0.652) and SP3 (*p* = 0.605) were not (Figure S4C,D). Collectively, these results demonstrate that PSOSP can accurately determine the classification of prophages in terms of SOS dependency (Table S4).

## Systematic analysis of SiPs and SdPs in bacterial genomes

**FIGURE 2:**
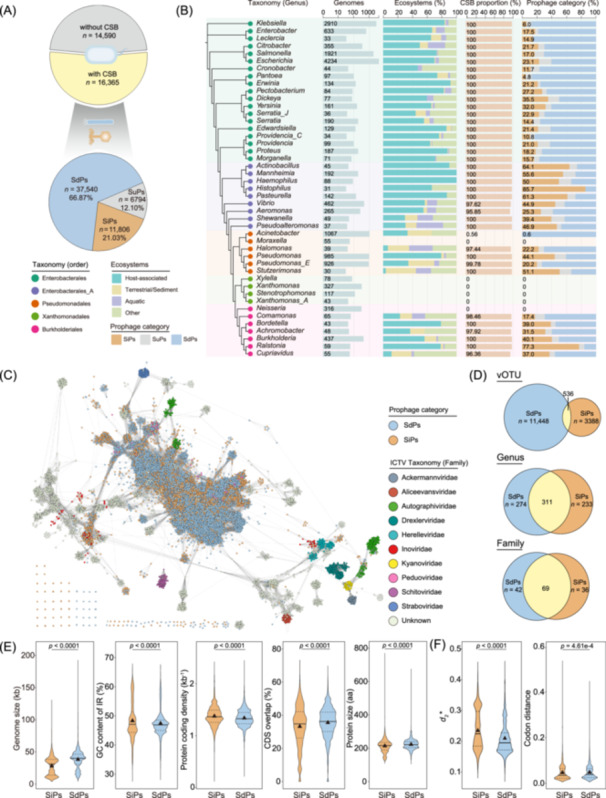
Widespread existence of SOS‐independent prophages (SiPs) and the comparison between SiPs and SOS‐dependent prophages (SdPs). (A) Distribution of bacteria with canonical SOS boxes (CSBs) and non‐CSBs (top panel) and the proportions of SiPs, SdPs, and SOS‐uncertain prophages (SuPs) among prophages (bottom panel). (B) Distribution of prophage types in Gammaproteobacteria. For clarity, only genera with ≥30 genomes are shown. (C) The protein‐sharing network of SiPs and SdPs using vConTACT v2.0. Nodes represent vOTUs, while edges indicate shared proteins. Nodes are depicted in the color representing viral operational taxonomic unit (vOTUs) of SiPs (orange) and SdPs (blue). Taxonomically assigned viruses from ICTV were included for reference. (D) Proportions of unique and shared SiPs and SdPs at different taxonomic ranks. The proportions of unique and shared SiPs and SdPs are illustrated. The numbers corresponding to each segment at each taxonomic rank (vOTU, genus, and family) are indicated in the pie charts. Comparison of genomic features (E) and phage‐host dissimilarity (F) between SiPs and SdPs. Codon distance was characterized by calculating the cosine distance between codon frequency vectors (Dc(phage, host)) for prophage and host sequences. Statistical significance was assessed using a two‐sided unpaired Welch's *t* test, with *p* values displayed above each plot. In the violin plots, solid and dashed black lines represent the median and the quartiles, respectively. Black triangles denote the means.

To understand the distribution of SiPs and SdPs in bacterial genomes, we applied PSOSP to analyze 49,333 bacteria with complete genome sequences from the NCBI RefSeq database [19], of which 30,955 bacterial genomes contained complete prophages with defined boundaries (*n* = 86,548) following rigorous filtration steps (Figure S5 and Supplemental Note 2). We found that 53% (*n* = 16,365) of these bacteria harbored CSBs (Figure 2A). Among the top 10 most abundant CSB‐carrying bacterial species, we performed pairwise interspecies comparisons for both *HI_C1_* and *HI_C2_* thresholds, yielding 45 comparisons for each threshold. Approximately 90.0% of these comparisons showed statistically significant differences within each threshold category, indicating genome‐specific *HI* patterns (Figure S6 and Supplemental Note 3). The majority (99.34%) of CSB‐carrying bacteria belonged to Gammaproteobacteria, while only a small fraction was found in other bacterial classes (Figure S7). Analysis of the *HI*_min_ values of these Gammaproteobacteria‐associated prophages revealed significant differences (*p* < 0.0001) between SiPs (average = 17.85) and SdPs (average = 7.53) (Figure S7). Among the prophages identified by PSOSP, 11,806 were classified as SiPs (21.03%), 37,540 as SdPs (66.87%), and 6794 as SuPs (12.10%). Notably, within Gammaproteobacteria, SiPs were found widely distributed across multiple genera (Figure 2B and Supplemental Note 4) with diverse induction mechanisms reported (Supplemental Note 5). In certain genera, such as *Histophilus*, *Ralstonia*, *Actinobacillus*, and *Pasteurella*, their proportion exceeded 60%. Additionally, no CSBs were detected in bacterial genomes of *Xylella*, *Xanthomonas*, *Stenotrophomonas*, and *Xanthomonas_A*, while no LexA was identified in *Neisseria* genomes. As a result, prophage types were not predicted for these genera (Figure 2B and Figure S8, Table S5), reflecting inherent limitations in the current applicability of PSOSP (Supplemental Note 6 and Discussion 1, Figure S9). Overall, different prophages within the same strain often exhibited distinct induction types; however, in some genera, such as *Ralstonia* and *Pasteurella*, coexisting prophages tended to share the same induction mode (Figure S10).

Subsequently, we analyzed the taxonomic and genomic characteristics of SiPs and SdPs to delineate the features of these two prophage groups. Overall, SiPs and SdPs exhibit both distinct clustering and overlapping patterns, with similarities to multiple viral families recognized by the ICTV (Figure 2C). The weighted gene repertoire relatedness (wGRR) analysis yielded consistent results, showing that the internal similarities within SiPs and SdPs were significantly higher than the similarities between them (*p* < 0.0001) (Figure S11). At different taxonomic levels, the degree of shared features between SiPs and SdPs increased with higher taxonomic ranks (from viral operational taxonomic unit (vOTU) to family), indicating that their divergence primarily occurs at the species level or below (Figure 2D). The clustering feature may suggest the potential for mutual conversion between certain SiP and SdP groups (Supplemental Discussion 2 and Figure S12). Across various environments, SdPs were generally more prevalent than SiPs, with SiPs accounting for 15.0%–29.6% of prophages, whereas SdPs ranged from 54.0%–74.5% (Figure S13). Further comparative enrichment analysis indicated that SiPs exhibited a preferential distribution in plant‐related, some terrestrial/sediment and aquatic environments (Figure S14). In terms of the functional gene composition, SiPs exhibited a higher proportion of tail‐related proteins compared to SdPs, but a lower proportion of genes related to nucleotide metabolism and transcription regulation (Figure S14). Compared to SdPs, SiPs have a significantly lower proportion of CDS overlap (PCO) (*p* < 0.0001) and smaller protein sizes (*p* < 0.0001), but higher GC content of intergenic region (IR) (*p* < 0.0001) and protein‐coding density (PCD) (*p* < 0.0001) (Figure 2E and Table S6). This difference in GC content and PCD may be related to evolutionary divergence between SiPs and SdPs (Supplemental Discussion 2). Notably, SiPs exhibited significantly smaller genomes than SdPs (*p* < 0.0001) and displayed dissimilar distribution patterns in both genome size (*p* < 0.0001, Kolmogorov–Smirnov (K–S) test) and completeness (*p* < 0.0001, K–S test), yet maintained similar distributions of phage‐specific functions (*p* = 0.99, K–S test) (Figure S14).

Besides, differences were also observed between the hosts of SiPs and SdPs. Specifically, SiPs tend to infect bacteria with lower PCD (*p* < 0.0001) and PCO (*p* < 0.0001), but higher GC content (*p* < 0.0001) and protein size (*p* < 0.0001) compared to SdPs (Figure S15 and Table S7). Additionally, the proportion of fast‐growing bacteria among SiP hosts was generally lower than that of SdP hosts. Furthermore, the optimal growth temperature and salinity of SiP hosts were lower, while their optimal growth pH was higher (Figure S15). Notably, SiPs exhibited greater nucleotide feature divergence from their hosts compared to SdPs (Figure 2F and Figure S16, Table S8), suggesting lower compatibility with their hosts. However, in terms of codon distance, SiPs showed higher similarity to their hosts than SdPs. In summary, these findings highlight the unique genomic features of SiPs and SdPs, underscoring their distinct evolutionary trajectories and ecological preferences.

In conclusion, we developed a novel bioinformatics tool PSOSP to predict prophage induction modes by analyzing the *HI* of LexA protein binding to target DNA, classifying prophages into SdPs or SiPs. PSOSP was experimentally validated to accurately distinguish SdPs from SiPs. We discovered that SiPs were widely distributed within bacterial genomes and exhibited distinct genomic features compared to the more well‐studied SdPs. Correspondingly, the hosts of these two prophage types are hypothesized to differ in their physiological characteristics. These PSOSP‐enabled findings provide not only novel insights into diverse induction mechanisms but also a critical methodology for future studies on phage–host interactions and prophage isolation strategies.

Detailed procedures for experimental methods, data collection, bioinformatic and statistical analysis approaches are available in the Supporting Information. The bacterial strains, plasmids, and primers used in this study are listed in Tables S9 and S10.

## AUTHOR CONTRIBUTIONS

**Yali Hao**: Methodology; data curation; validation; formal analysis; supervision; writing—review and editing; visualization; project administration. **Mujie Zhang**: Methodology; software; data curation; validation; formal analysis; supervision; writing—review and editing; visualization. **Xinjuan Lei**: Formal analysis; methodology. **Chengrui Zhu**: Formal analysis. **Taoliang Zhang**: Funding acquisition; supervision. **Yanping Zheng**: Supervision; funding acquisition. **Xiang Xiao**: Supervision; funding acquisition. **Huahua Jian**: Conceptualization; data curation; methodology; supervision; funding acquisition; writing—original draft; writing—review and editing; project administration.

## CONFLICT OF INTEREST STATEMENT

The authors declare no conflicts of interest.

## ETHICS STATEMENT

No animals or humans were involved in this study.

## Supporting information


**Figure S1:** Classification of *HI* for all PSBs in *E. coli* K12 using different clustering methods.
**Figure S2:** Validation of the SOS‐dependency of prophage SW1 in *S. piezotolerans* WP3.
**Figure S3:** Distribution of PSBs and *HI* values for experimentally validated eight SdPs and two SiPs.
**Figure S4:** Validation of the SOS‐dependency of the three prophages in *S. psychrophila* WP2.
**Figure S5:** Genome completeness and length distribution of prophages from different sources.
**Figure S6:** Comparison of *HI* values across bacterial genomes of different taxonomic levels.
**Figure S7:** Distribution of *HI* clusters in bacteria.
**Figure S8:** Genera retained and filtered out in the analysis of bacterial genomes from the RefSeq database (Release 226) by PSOSP.
**Figure S9:** Performance evaluation of PSOSP on host and phage genomes of varying quality.
**Figure S10:** Consistency of regulatory patterns among multiple prophages in polylysogenic strains.
**Figure S11:** Viral similarity analysis based on wGRR.
**Figure S12:** Representative case of mutual conversion between SiP and SdP.
**Figure S13:** Distribution of SiPs, SuPs, and SdPs across different environments.
**Figure S14:** Comparison of genome size, host distribution, environmental distribution, and protein function categories between SdPs and SiPs.
**Figure S15:** Comparative analysis of genomic and growth characteristics between SiPs hosts and SdPs hosts.
**Figure S16:** Comparative analysis of nucleotide frequency divergence between SiPs and SdPs and their respective hosts.

**Table S1:** Clustering results of seven methods for *HI* of potential SOS boxes (PSB) in *E. coli* K12.
**Table S2:** Classification thresholds derived from clustering results.
**Table S3:** Performance comparison of seven clustering methods in classifying 24 validated SOS boxes in *E. coli* K12.
**Table S4:** Experimentally validated induction‐mode bacteriophages and hosts in this study.
**Table S5:** Genera retained and filtered out in analysis of bacterial genomes from the RefSeq database (Release 226) by PSOSP.
**Table S6:** Detailed data on genomic characterization of prophages.
**Table S7:** Detailed data on genomic characterization of bacteria.
**Table S8:** Nucleotide frequency divergence between SiPs and SdPs and their respective hosts.
**Table S9:** Bacterial strains and plasmids used in this study.
**Table S10:** Primers used in this study.

## Data Availability

The data that support the findings of this study are openly available at https://github.com/mujiezhang/SOS-independent-prophages. All assembled prokaryotic genomes (*n* = 383,026) used in this study were collected from publicly available databases including the NCBI RefSeq (Release 226) [20]. The VIP‐Seq data generated in this study have been deposited in the European Nucleotide Archive (ENA) at EMBL‐EBI under accession number PRJEB91164 (https://www.ebi.ac.uk/ena/browser/view/PRJEB91164) and are also available in the National Omics Data Encyclopedia (NODE) data under the Project ID OEP00006037 (https://www.biosino.org/node/project/detail/OEP00006037) & OEP00006346 (https://www.biosino.org/node/project/detail/OEP00006346). The PSOSP code is available under an open‐source license on GitHub (https://github.com/mujiezhang/PSOSP). A web server implementing PSOSP is accessible at https://vee-lab.sjtu.edu.cn/PSOSP/. Additionally, the code used to generate the figures presented in the manuscript can be downloaded from GitHub (https://github.com/mujiezhang/SOS-independent-prophages). Supplementary materials (methods, figures, tables, graphical abstract, slides, videos, Chinese translated version, and update materials) can be found in the online DOI or iMeta Science http://www.imeta.science/.
