# Supplementary material for: PSOSP uncovers pervasive SOS‐independent prophages with distinct genomic and host traits in bacterial genomes
Source: Imeta. 2025 Aug 18;4(5):e70073. doi: 10.1002/imt2.70073 (PMC12527982; doi:10.1002/imt2.70073)
Supplement: Supplementary file 1 — Figure S1: Classification of HI for all PSBs in E. coli K12 using different clustering methods. Figure S2: Validation of the SOS‐dependency of prophage SW1 in S. piezotolerans WP3. Figure S3: Distribution of PSBs and HI values for experimentally validated eight SdPs and two SiPs. Figure S4: Validation of the SOS‐dependency of the three prophages in S. psychrophila WP2. Figure S5: Genome completeness and length distribution of prophages from different sources. Figure S6: Comparison of HI values across bacterial genomes of different taxonomic levels. Figure S7: Distribution of HI clusters in bacteria. Figure S8: Genera retained and filtered out in the analysis of bacterial genomes from the RefSeq database (Release 226) by PSOSP. Figure S9: Performance evaluation of PSOSP on host and phage genomes of varying quality. Figure S10: Consistency of regulatory patterns among multiple prophages in polylysogenic strains. Figure S11: Viral similarity analysis based on wGRR. Figure S12: Representative case of mutual conversion between SiP and SdP. Figure S13: Distribution of SiPs, SuPs, and SdPs across different environments. Figure S14: Comparison of genome size, host distribution, environmental distribution, and protein function categories between SdPs and SiPs. Figure S15: Comparative analysis of genomic and growth characteristics between SiPs hosts and SdPs hosts. Figure S16: Comparative analysis of nucleotide frequency divergence between SiPs and SdPs and their respective hosts. [file IMT2-4-e70073-s002.docx]

**Supporting information to**

**PSOSP uncovers pervasive SOS-independent prophages with distinct genomic and host traits in bacterial genomes**

**Running title**: Pervasive SOS-independent prophages in bacterial genomes

Yali Hao^1, 2#^, Mujie Zhang^1, 2#^, Xinjuan Lei^1, 2^, Chengrui Zhu^1^, Taoliang Zhang^2^, Yanping Zheng^3^, Xiang Xiao^1, 2^, Huahua Jian^1, 2*^

^1^State Key Laboratory of Microbial Metabolism, Joint International Research Laboratory of Metabolic & Development Sciences, School of Life Sciences and Biotechnology, Shanghai Jiao Tong University, Shanghai 200240, China

^2^Yazhou Bay Institute of Deepsea Science and Technology, Hainan Research Institute, Shanghai Jiao Tong University, Sanya 572024, China

^3^Center for Precision Medicine, The First Affiliated Hospital of Xiamen University, School of Medicine, Xiamen University, Xiamen 361003, China

^#^These authors contributed equally: Yali Hao, Mujie Zhang

*Correspondence: [jiandy@sjtu.edu.cn](mailto:jiandy@sjtu.edu.cn) (Huahua Jian)

**Supplemental Notes**

**Supplemental Note 1. Selection of PSB clustering method, determination of *HI* thresholds, and prophage classification**

The distribution of Heterology Index (*HI*) values for all potential SOS boxes (PSBs) identified in *Escherichia coli* K12 exhibits a clear bimodal pattern, with one peak centered at a low *HI* value (7.34) and the other at a high *HI* value (22.32). Among the 24 experimentally validated PSBs, 19 that are capable of binding LexA fall within the low-*HI* peak, while the remaining 5 that cannot bind LexA fall within the high-*HI* peak. This observation suggests that accurate clustering of PSBs into these two peaks could be used to predict the LexA-binding potential of novel PSBs.

To accurately cluster all PSBs and derive an appropriate *HI* threshold, we applied seven unsupervised clustering algorithms from the scikit-learn Python package (v1.6.1) [1] including Mean Shift, K-means, Birch, Gaussian Mixture, Bayesian Gaussian Mixture, Agglomerative Clustering, and Spectral Clustering. Each method aimed to cluster *E. coli* K12 PSBs into two biologically meaningful classes: low-*HI* (high-affinity LexA binders) and high-*HI* (low-affinity non-binders). We defined the mean of the *HI* values at the boundary between these two classes as the central demarcation point *HI_cm_*. Given the complexity of protein-DNA interactions and the possibility of overlap between categories, we introduced a buffer zone: *HI_C1_* (95% of *HI_cm_*, upper threshold of the low-*HI* category) and *HI_C2_* (105% of *HI_cm_*, lower threshold of the high-*HI* category). Validation against the 24 benchmark PSBs demonstrated that only thresholds derived from Mean Shift clustering achieved perfect classification accuracy (Table S3 and Figure S1), correctly distinguishing all binding-competent from non-binding PSBs.

Prophages were classified based on the minimal *HI* value (*HI_min_*) among their constituent PSBs: SOS-dependent prophages (SdPs): *HI_min_* < *HI_C1_* (contains high-affinity PSB); SOS-independent prophages (SiPs): *HI_min_* > *HI_C2_* (contains low-affinity PSB); and SOS-uncertain prophages (SuPs): *HI_C1_* ≤ *HI_min_* ≤ *HI_C2_* (indeterminate binding status).

**Supplemental Note 2. Evaluation of the effectiveness of the prophage quality control strategy**

To assess the effectiveness of our quality control (QC) strategy (see Methods for details), we compared the genome completeness of three prophage sets: those that passed QC (“qualified”), those that did not (“unqualified”), and a publicly available prophage database, Prophage-DB [2]. As shown in Figure S5, the qualified prophages exhibited significantly higher genome completeness (average (avg) = 82.2%) than both unqualified prophages (avg = 43.1%) and entries in Prophage-DB (avg = 19.3%) (*p* < 0.0001). Notably, 67.4% of the qualified prophages had completeness levels above 90%. In terms of genome length, the qualified prophages were also significantly longer (avg = 35.7 kb) compared to the unqualified (avg = 18.5 kb) and Prophage-DB prophages (avg = 13.1 kb) (*p* < 0.0001), indicating that our QC pipeline effectively filters out fragmented or partial prophage sequences.

Interestingly, within the qualified prophage set, a subset (*n* = 10,572; 18.8%) was predicted by CheckV to be of low quality (completeness < 50%). Manual inspection revealed that these low-quality cases were due to limitations in CheckV’s estimation model. Specifically, CheckV assesses completeness by comparing the genome length of the query virus with those of reference genomes within the same genus or family [3]. However, highly novel viruses may lack sufficient similarity to existing references, leading to inaccurate completeness estimates. For example, SiPs-1827 (7427 bp) was predicted by CheckV to have low completeness (48.07%). However, manual validation showed that it shares high homology with *Vibrio* phage VALG_phi8 (MN690600, 7311 bp), which has a complete genome sequence, with 99.69% identity and 91.79% coverage. These two sequences belong to the same vOTU, yet CheckV also predicted VALG_phi8 to have low completeness (49.1%). Thus, our QC strategy not only effectively removes incomplete prophages but also helps retain complete but novel viral genomes that may be incorrectly flagged by automated tools like CheckV.

**Supplemental Note 3. Comparability of *HI* values across bacterial genomes**

We systematically analyzed *HI* classification thresholds in the genomes of the 10 most abundant bacterial species and genera (Figure S6). For each species, we performed pairwise interspecies comparisons for both *HI_C1_* and *HI_C2_* thresholds, resulting in 45 comparisons per threshold category. Approximately 90.0% of these interspecies comparisons showed statistically significant differences within each threshold category. Similar analyses at the genus and family levels revealed significant differences in 94.4% of intergenus and 90.0% of interfamily comparisons. These variations likely stem from sequence divergence in SOS boxes across taxonomic units [4]. These findings suggest that *HI* is highly genome-specific and therefore should not be directly compared across different bacterial genomes.

**Supplemental Note 4. Widespread distribution and taxonomic preference of SiPs**

Our analysis was based on 49,333 bacterial genomes labeled as “Complete genome” in the NCBI RefSeq database (source: *https://ftp.ncbi.nlm.nih.gov/genomes/refseq/bacteria/assembly_summary.txt*). According to the official RefSeq definition, a “Complete genome” refers to: “*All chromosomes are gapless and contain runs of nine or less ambiguous bases (Ns), there are no unplaced or unlocalized scaffolds, and all the expected chromosomes are present (i.e., the assembly is not noted as having partial genome representation). Plasmids and organelles may or may not be included in the assembly, but if they are present, the sequences are gapless. The chromosome sequences may not be fully complete from telomere-to-telomere (T2T).*” These genomes span 135 bacterial classes, representing high taxonomic diversity. Using PSOSP, we successfully identified prophage induction types in 15,493 genomes. Notably, Gammaproteobacteria accounted for the majority of these predictions, involving 15,474 genomes across 187 genera. Among these, SiPs were identified in 7721 genomes (49.9%) and 141 genera (75.4%), highlighting their widespread occurrence.

To evaluate whether our genome selection strategy (*i.e.*, using only complete genomes) or any inherent database bias could have artificially amplified the dominance of Gammaproteobacteria in our results, we performed the following comparative analysis. In the full NCBI RefSeq prokaryotic genome dataset (383,026 genomes), Gammaproteobacteria account for 43.2% (165,596/383,026). By comparison, in our study’s dataset of 49,333 complete genomes, Gammaproteobacteria represent 41.9% (20,671/49,333). These two proportions are very close, which is insufficient to explain the observation that nearly all predicted SOS boxes originate from Gammaproteobacteria. Therefore, we conclude that our genome selection strategy did not introduce a bias toward Gammaproteobacteria. We acknowledge that the dominance of Gammaproteobacteria in our predictions may partially reflect an existing bias in public databases, where genomes from this class are overrepresented. However, we believe that the observed result is also driven by a biological factor—namely, the natural and widespread presence of LexA proteins and canonical SOS boxes in Gammaproteobacteria—rather than by subjective selection bias in our dataset. We further speculate that the relatively late evolutionary divergence of Gammaproteobacteria [5], along with the late emergence of canonical SOS boxes in the evolutionary timeline [4−7], has led to the retention of highly conserved and structurally stable SOS box motifs in this group. As a result, these canonical motifs are more readily detected by PSOSP, potentially explaining their enrichment in our results.

It is important to emphasize that the taxonomic representation in the current RefSeq database remains unbalanced, and many microbial groups are still far from being comprehensively sampled. As more high-quality genomes from underrepresented microbial taxa become available in the future, the distributions of LexA-encoding genes, SOS boxes, as well as SdPs and SiPs, will need to be reevaluated in a broader and more balanced taxonomic context.

**Supplemental Note 5. Diverse SOS-Independent mechanisms of prophage induction**

In addition to the classical SOS-dependent pathway, prophages can be induced through various SOS-independent mechanisms, highlighting the complexity of prophage regulatory networks [8,9]. Currently known SOS-independent induction mechanisms include: (1) spontaneous induction: for instance, in the degenerated prophage CGP3 of *Corynebacterium glutamicum*, approximately 30% of spontaneous induction events have been demonstrated to be RecA-independent, indicating the presence of endogenous induction pathways independent of the SOS response [10]; (2) environmental stress-induced induction: in some variants of bacteriophage λ (*e.g.*, CI857), heat stress can lead to inactivation of the repressor protein, thereby triggering entry into the lytic cycle [11]. Similarly, in phage λ^imm434^, salt stress (*e.g.*, high concentrations of Na⁺ or K⁺) reduces the binding affinity between the repressor and DNA, promoting the lytic switch [12]; and (3) quorum-sensing signal molecule-induced induction: in the case of *Vibrio* phage VP882, a VqmA homolog encoded by the phage can bind to the host-produced quorum-sensing molecule DPO in *Vibrio cholerae*, thereby activating the Qtip protein to sequester the repressor and initiate lytic development [13]. It is important to note that these mechanisms are often phage-specific. Broader, more generalizable principles underlying SOS-independent prophage induction remain to be systematically explored and elucidated.

**Supplemental Note 6. Performance of PSOSP on bacterial genomes of varying quality**

To evaluate the performance of PSOSP on incomplete genomes with varying quality—such as metagenome-assembled genomes (MAGs)—we conducted a systematic assessment: First, 1000 bacterial genomes were randomly selected from the dataset used in this study. Following the mock genome construction strategy implemented in CheckM (see Methods for details), we generated 3000 mock host genomes with varying completeness (50−100%) and contamination levels (0−20%). Similarly, based on 3599 prophages identified in these genomes, we created 10,797 mock viral genomes using the CheckV-based simulation approach (see Methods for details), with completeness ranging from 50−100% and contamination from 0−50%. Then, we evaluated the consistency between PSOSP predictions on the mock genomes and those from the original genomes. The results (Figure S9) showed: (1) Host genome completeness was significantly correlated with prediction success rate (*R^2^* = 0.66, *p* < 0.0001). This is likely because low-completeness genomes tend to lack LexA-encoding genes; (2) Viral genome completeness does not significantly affect prediction success rate (*R²* = 0.00015, *p* = 0.93), but is strongly correlated with prediction precision (*R²* = 0.84, *p* < 0.0001), as incomplete viral genomes are more likely to lack detectable SOS boxes; and (3) Contamination levels in both host and viral genomes do not significantly affect prediction precision (*p* = 0.084 and 0.31, respectively).

Based on these results, we recommend using bacterial and viral genomes with ≥ 90% completeness as input to ensure accurate predictions. To assist users in assessing the quality of input data, we integrated CheckV into PSOSP. Quality annotations are added to the prediction results: predictions classified as SdPs are labeled as “high-quality”, while those classified as SuPs or SiPs are categorized based on viral genome completeness: ≥ 90% (high-quality), 50−90% (medium-quality), and < 50% (low-quality).

**Supplemental Discussions**

**Supplemental Discussion 1. The limitation and perspective of PSOSP**

Although the PSOSP tool demonstrates high accuracy in predicting prophage induction mechanisms, several areas warrant improvement to enhance its predictive capabilities. First, PSOSP primarily focuses on groups with the canonical SOS box (N_2_-CTG-N_10_-CAG-N_2_), while without considering those harboring non-canonical SOS boxes (Table S5). Emerging evidence reveals substantial variation in LexA binding motifs: *Xanthomonas axonopodis* pv. *citri* possesses TTAGN_11_CTAA [14]; *Pseudomonas putida* contains both *E. coli*-like LexA boxes and distinct binding sequences GTAC-N_4_-GTRC [15,16]. Systematic evaluation of prophages with non-canonical SOS boxes would significantly expand PSOSP’s applicability. Furthermore, the completeness of both the host and prophage genomes significantly influences the precision of PSOSP (Figure S9 and Supplemental Note 6). Reliable prediction typically requires genome completeness exceeding 90% for both the host and prophage. This requirement limits the applicability of PSOSP to host-prophage pairs derived from low-quality metagenomic assemblies. Notably, prophages with PSBs exhibiting intermediate *HI* values (transitional range between SdPs and SiPs) are classified as SuPs by PSOSP. Lastly, PSOSP assigns each phage exclusively as either SiP or SdP, yet experimental evidence suggests that some phages may possess dual induction mechanisms. For instance, VP882 undergoes induction through both quorum sensing and SOS pathways [17]. Future enhancements integrating diverse induction mechanism data and algorithmic optimization would enable PSOSP to accurately determine SuPs’ induction patterns, thereby significantly expanding its predictive scope.

**Supplemental Discussion 2. Potential conversion and evolutionary dynamics between SiPs and SdPs**

Analysis of the protein similarity networks of SiPs and SdPs reveals a coexistence of “independent clustering” and “interwoven distribution” patterns, with increasing shared characteristics at higher taxonomic levels (Figure 2C,D). This phenomenon suggests the potential of mutual conversion between certain SiPs and SdPs groups. Considering that the primary criterion for classifying SiPs and SdPs is based on the comparison between *HI* of their PSBs and the corresponding *HI* threshold, such conversions are plausible: mutations in PSBs located within the intergenic regions of SdPs and SiPs could alter their *HI* values, thereby modifying their binding affinity to LexA and subsequently shifting their induction modes. In fact, the binding affinity of LexA is significantly influenced by the AT content of its binding sequences [18], indicating that mutational biases may drive these conversions. This hypothesis aligns with known evolutionary mechanisms of bacterial regulatory elements: bacterial transcriptional regulatory elements often exhibit high flexibility and rapid evolutionary diversification in response to environmental changes [19]; experimental evidence from de novo evolution of the *E. coli* *lac* promoter demonstrated that single nucleotide mutations can confer significant promoter activity to random DNA sequences, with subsequent mutations further enhancing similarity to conserved promoters [20]. In support of this, we identified potential cases of transition between SdPs and SiPs. For example, in *E. coli*, we discovered two nearly identical prophages, SdPs-30201 and SiPs-1110 (identity: 99.99%, coverage: 100%). Despite their minimal genomic differences, and with only a single nucleotide variation (T→C) in their SOS box sequences, their corresponding *HI* values differed (14.81 and 17.09), leading to their classification as an SdP and a SiP, respectively (Figure S12). This finding suggests that prophage induction modes may shift through minimal genetic changes, such as single-nucleotide substitutions. While these hypotheses were generated through the PSOSP tool, future molecular biology experiments will be required to validate the regulatory mode transitions of these prophages.

From the perspective of phage-host coevolution, the coupling between temperate phage induction factors and host physiological state benefits both parties: phages gain the ability to sense host survival status, enabling them to make decisions favorable to their own propagation, while hosts integrate prophages into their regulatory networks to optimize the control of their lifecycles, thereby minimizing metabolic burdens and mitigating the risk of cell lysis posed by these potentially detrimental “time bombs”. The distinct genomic features between SiPs and SdPs—particularly the observation that SiPs exhibit higher GC content and PCD than SdPs (Figure 2E)—offer insights into their coevolutionary dynamics with bacterial hosts. Specifically, given the widespread GC→AT mutational bias in bacterial genomes [21,22], the elevated GC content in SiPs suggests both strong mutational pressure and their early-stage coevolution with hosts, where AT-biased mutations have yet to accumulate substantially. This hypothesis is further supported by higher nucleotide divergence between SiPs and their hosts (Figure 2F). Moreover, the higher PCD of SiPs, along with their greater divergence from host genomes (Figures 2E and S15), further supports the notion that SiPs may be in an earlier phase of host adaptation. Alternatively, it may reflect an evolutionary preference for more compact genome structure under non-SOS regulatory mechanisms. We hypothesize that the choice of induction strategies in temperate phages may be governed by a complex interplay of factors, including the characteristics of phage regulatory elements, host regulatory networks, inter-phage competition, and relevant environmental signals. Therefore, the potential interconversion between SiPs and SdPs groups and its underlying mechanisms remains to be elucidated through further exploration.

**Supplemental Methods**

**Identification of LexA and SOS boxes**

The amino acid sequences of LexA protein (*n* = 458) were extracted from UniProtKB/Swiss-Prot [23] and used to construct a reference LexA protein dataset. Prodigal was used to determine the open reading frames (ORFs) of prophages and their hosts with the parameter “-p meta”. LexA proteins encoded by prokaryotic genomes were identified by BLASTp using DIAMOND against the reference LexA protein dataset, with a cut-off value of e < 10^-10^, an identity ≥ 30% [24,25]. The position-specific scoring matrix (PSSM) for 19 classical SOS boxes of *E. coli* K12, as previously described [26], was generated by MEME (v5.5.5) [27] with parameters “-dna -mod oops -pal -w 20”. The putative promoter region (-300 bp to +50 bp of the predicted start codon) of *lexA* gene was scanned by FIMO (v5.5.5) [28] using the PSSM with default parameters. Only genomes containing both *lexA* gene and classical SOS box in *lexA* promoter region were considered for further analysis. All possible SOS boxes were identified with the conserved motif (N_2_-CTG-N_10_-CAG-N_2_) using an in-house script in the putative promoter regions of all genes. Genes in the same orientation with an intergenic distance < 20 bp were considered to constitute an operon.

**Heterology Index (*HI*) calculation and prophage categorization**

Heterology Index (*HI*) was calculated as defined by Lewis et al [26]. *HI* values of potential SOS boxes in each genome were clustered using the mean shift clustering algorithm from scikit-learn (v1.6.1) [1]. We defined the mean of *HI* values at the boundary between these two classes as the central demarcation point *HI_cm_*. To identify putative SOS boxes within proviral genomes, a 20-bp sliding window analysis (with a 1-bp step) was performed across the predicted promoter regions. The window exhibiting the minimum *HI* value was identified as the potential SOS box and annotated as *HI_min_*.

Prophages were classified into three categories based on quantitative thresholds: (1) SOS-dependent prophages (SdPs): *HI_min_* < *HI_C1_*; (2) SOS-independent prophages (SiPs): *HI_min_* > *HI_C2_*; and (3) SOS-uncertain prophages (SuPs): *HI_C1_* ≤ *HI_min_* ≤ *HI_C2_*. Here, *HI_C1_* = *HI_cm_* - 0.05×(*HI_cm_* - *HI_hostmin_*) and *HI_C2_* = *HI_cm_* + 0.05×(*HI_hostmax_* - *HI_cm_*). *HI_hostmax_* and *HI_hostmin_* respectively represent the maximum and minimum *HI* values across the entire prokaryotic host genome.

**Bacterial strains and growth conditions**

The bacterial strains and plasmids used in this study are listed in Table S9. *E. coli* strains were cultured in lysogeny broth (LB) medium (10 g/L tryptone, 5 g/L yeast extract, 10 g/L NaCl) and incubated at 37 °C with shaking at 220 rpm. *Shewanella* strains were cultured in modified 2216E marine medium (5 g/L tryptone, 1 g/L yeast extract, 0.1 g/L FePO_4_, 34 g/L NaCl) at 15 °C or 20 °C with shaking at 220 rpm. Media were supplemented with kanamycin (50 μg/mL), when needed.

**RT-qPCR measurements**

The untreated *S. piezotolerans* WP3 and the *lexA* gene deletion mutant strain WP3Δ*lexA* were inoculated into 2216E medium. For both MMC and UV treatments, the WP3 cells were cultivated to an OD_600_ of 0.3, and then treated with 0.5 μg/mL mitomycin C (MMC) for 3 h or exposed to UV (25 J/m²) for 30 s. The cells were then harvested by centrifugation, resuspended in 2216E medium, and grown for 1 h. Total RNA was extracted from the MMC- and UV-treated, untreated WP3, and untreated WP3Δ*lexA* cell cultures using the RNAprep Pure Cell/Bacteria kit (TIANGEN Biotech, Beijing, China). DNA contamination was removed by treating RNA samples with DNase I at 37 °C for 1 h. The RNA was reverse transcribed into complementary DNA (cDNA) using the RevertAid First Strand cDNA Synthesis Kit (Fermentas, Maryland, USA). Primer pairs for RT-qPCR were designed with Primer Express software (v3.0.1, Applied Biosystems, CA, USA) (Table S10). PCR amplification was performed on a 7500 real-time PCR system (Applied Biosystems, CA, USA) using 20 μl reaction mixtures containing 1× SYBR Green I Universal PCR Master Mix (Applied Biosystems), 0.5 μM of each primer, and 1 μl of cDNA template.

**Overexpression and purification of LexA protein**

The *lexA* gene from *S. piezotolerans* WP3 was synthesized and cloned into the pET28a plasmid (Tsingke Biotech, Beijing, China). Recombinant plasmids were introduced into *E. coli* DH5α cells, which were selected on LB plates with kanamycin (50 μg/mL) and confirmed by PCR and DNA sequencing. The recombinant plasmids were then extracted and transformed into *E. coli* BL21 (DE3). The cells were cultured in LB broth at 37 °C, and protein expression was induced with IPTG (0.8 mM) when the OD_600_ reached 0.6. The cultures were incubated at 15 °C for 16 h. Cells were harvested by centrifugation, resuspended in binding buffer containing 500 mM NaCl, 10% glycerol, 20 mM Tris-HCl (pH 8.0), and sonicated on ice. The supernatant was collected by centrifugation and purified using Ni-TED Sepharose 6FF (Sangon Biotech, Shanghai, China) following the manufacturer’s instructions. The protein was eluted in a buffer containing 500 mM NaCl, 10% glycerol, 300 mM imidazole, and 20 mM Tris-HCl (pH 8.0). The eluted protein solution was subsequently buffer-exchanged into the target experimental buffer (150 mM NaCl, 5% glycerol, 20 mM Tris-HCl, pH 8.0) using Amicon Ultra centrifugal filter devices (10 kDa molecular weight cut-off (MWCO), Merck Millipore) according to the manufacturer’s protocol. Protein purity was assessed by SDS-PAGE (12% acrylamide) and quantified using the Bradford Protein Assay Kit (Sangon Biotech, Shanghai, China).

**Electrophoretic mobility shift assay (EMSA)**

DNA probes for *recA* and 100-bp-long DNA sequences containing potential SOS boxes (PSBs) were amplified from the genomic DNA of WP3. The mutations in PSB-1, 2, and 3 were amplified from the corresponding plasmids. For example, PSB-1 harboring mutation 1 (PSB-1-M1) was amplified from the plasmid pUC57-PSB-1-M1 using 6-FAM-labeled primers (Table S10). The 6-FAM-labeled DNA fragments were purified and quantified using the Qubit 4.0 fluorometer (Thermo Fisher Scientific, MA, USA). For binding reactions, 0.2 pmol of DNA probes were incubated with varying amounts of purified protein at 25 °C for 30 min. Binding reactions were conducted in 20 μl mixtures containing 40 mM KCl, 12.5 mM Tris (pH 7.5), 125 μM MnCl_2_, 1.25 mM MgCl_2_, 1.25% glycerol, and 0.5 mM DTT. Protein-DNA complexes were resolved on 6% non-denaturing polyacrylamide gels and visualized using a gel imaging system (Clinx, Shanghai, China).

**Virion Induction Profiling Sequencing (VIP-Seq)**

Prophages in the supernatants of *S. psychrophila* WP2 were identified and quantified using Virion Induction Profiling Sequencing (VIP-Seq) as previously described [29]. WP2 cultures were cultivated in 2216E medium at 15 °C with shaking at 220 rpm until reaching stationary phase. Supernatants were collected via centrifugation (8000 × g for 5 min) and filtered through 0.22 μm filters (Merck Millipore, Ireland). To concentrate low-titre prophages, 30 mL of supernatant was processed using 30 kDa Amicon filters (Merck Millipore, Ireland) to < 1 mL, followed by addition of SM buffer (50 mM Tris-HCl, 8 mM magnesium sulfate, 100 mM sodium chloride, and 0.01% gelatin (w/v, pH 7.5)) to a final volume of 1.2 mL. DNase I (25 units/mL) and RNase A (25 μg/mL) were used to degrade non-encapsulated nucleic acids. Phage DNA was then extracted following treatment with EDTA (50 µM), SDS (0.1%), and Proteinase K (1 mg/mL) at 55 °C. The extracted DNA was purified using the Clean & Concentrator-5 kit (Zymo Research, USA) and quantified with a Qubit 2.0 Fluorometer (Thermo Fisher Scientific, MA, USA).

Sequencing libraries were prepared using the TruSeq DNA kit (Illumina, USA) and sequenced on the NovaSeq platform to generate 150-bp paired-end reads. Raw reads were trimmed using fastp (v0.23.4) [30], after which the reads were mapped to the WP2 genome using BWA (v0.7.18) [31]. Active prophage regions were identified through read coverage visualization in Integrative Genomics Viewer (IGV) (v2.17.4) [32]. The titre of each induced prophage was estimated using the formula:

$$\mathrm{induced} prophage/mL=\frac{m_{\mathrm{DNA}}\times N_{A}}{M_{\mathrm{nt}}\times L_{\mathrm{prophage}}}\times\frac{r_{\mathrm{pp}}}{r_{\mathrm{tot}}}$$

where *m_DNA_* is the total mass of eluted DNA adjusted per 1 mL overnight bacterial culture, *N_A_* is Avogadro’s constant, *M_nt_* is the average molar mass of a DNA nucleotide (617.96 g/mol/bp), *L_prophage_* is the length of the induced prophage genome, *r_pp_* is the number of reads mapped to the prophage region, and *r_tot_* is the total number of reads.

**Identification of prophages in prokaryotic genomes**

Prokaryotic genomes were retrieved from the RefSeq database (Release 226, *n* = 383,026) [33]. Only the genome assemblies labeled as “complete genome” (*n* = 49,333), which means all chromosomes are gapless and there are no unplaced or unlocalized scaffolds, and all the expected chromosomes are present [34], were used for further analysis. Prophages (*n* = 137,517) from complete bacterial genomes were predicted using geNomad (v1.8.0) [35] with default parameters. CheckV (v1.0.3) [3] was used to trim nonviral genomic regions. Then, the boundaries of prophages were delimited by searching for their attachment sites (*att*) as previously described [36]. First, a genomic region, referred to as the candidate prophages region, was obtained by extending 5 kb upstream and downstream of the trimmed prophage sequence. This region contained both potential prophage and host sequences at both ends. Then, the candidate prophage regions were aligned with all the other prokaryotic genomes in the RefSeq database using BLASTn (v2.13.0+) [37]. If two fragments (queries) flanking the prophage region could be aligned with two fragments (subjects) in another bacterium or archaeon genome and they were identified as homologous sequences (E-value < 1e-5), and showed at least 5 bp overlap at their ends (subjects), then they were identified as *attB* (in the subject genome), *attL*, and *attR* (in the query genome). Prodigal (v2.6.3) [38] was used to determine the open reading frames (ORFs) of boundary-defined prophages. Annotations were conducted using the PHROG database to retain viral functional protein categories, employing BLASTp using DIAMOND (v2.1.7.161) [39] with the “--more-sensitive” option. Only prophage sequences with identifiable *attL* and *attR* were considered as complete prophages and used for subsequent analyses.

For quality control, putative false positives of prophage sequences were further screened and excluded from the dataset based on the following predefined criteria: (1) viral sequences ≤ 4 kb in length [40]; (2) sequences flagged by CheckV as “high kmer_freq” and containing multiple Zot proteins, suggesting potential large-scale duplications [41−43]; (3) presence of complete genes within *attL* or *attR* regions, indicating potential false positives arising from large-scale repeats [44]; (4) detection of transposases on both genomic termini, suggesting chimeras formed between transposable elements and viral sequences [36,45]; and (5) absence of more than one type of protein associated with replication, lysis, or structural functions, being indicative of bacterial genomic fragments or incomplete viral remnants [46,47]. These stringent filtration steps effectively eliminated incomplete viral elements, significantly enhancing dataset reliability for downstream analyses (Figure S5 and Supplemental Note 2).

**Construction of phylogenetic trees**

GTDB-Tk (v2.3.2, database release 207_v2, ‘classify_wf’ function and default parameters) [48] was used to perform taxonomic annotation of each genome and reconstruct the maximum-likelihood phylogenetic tree based on 120 and 53 conserved single-copy genes in the bacterial and archaeal genomes, respectively. The unrooted phylogenetic tree of Gammaproteobacteria genomes was constructed by FastTree 2 (v2.1.11) [49] based on the maximum-likelihood algorithm with the JTT (Jones-Taylor-Thornton) model of amino acid evolution and visualized using tvBOT (v2.6) [50].

**Viral clustering and network analysis**

Complete prophages were clustered into virus operational taxonomic units (vOTUs) following MIUViG guidelines (95% average nucleotide identity (ANI); 85% aligned fraction (AF)) [51] using pipeline provided by CheckV [3]. In brief, prophages were first aligned in an all-versus-all BLASTn search with parameters “-task megablast -evalue 1e-5 -max target seqs 20000”. Then ANI and AF were computed, and the Leiden algorithm was performed to cluster sequences into vOTUs using anicalc.py and aniclust.py scripts provided by the website of CheckV (scripts available at https://bitbucket.org/berkeleylab/checkv/src/master/scripts/).

To cluster viral contigs at approximately the genus and family ranks, we used the method of gene sharing and average amino acid identity (AAI) referred to a previous study [52]. The longest viral contig from each species-level vOTU was used and all viral proteins were compared with each other using DIAMOND BLASTp with parameters “-e-value 1e-5 -max-target-seqs 10000 --query-cover 50 --subject-cover 50”. Then, AAI and percentage of shared proteins between each pair of contigs were calculated. Markov Cluster Algorithm (MCL) (v22-288) [53] was used for clustering and edges between contigs were filtered based on minimum AAI and gene sharing. For the family rank, an inflation factor of 1.2 was used, and connections with < 20% AAI or < 10% shared genes were excluded. For the genus rank, an inflation factor of 2.0 was applied and connections with < 50% AAI or < 20% shared genes were excluded.

Protein sharing network analysis of viral populations was performed by vConTACT (v2.0) [54]. In brief, the protein sequences of the vOTUs were grouped into protein clusters (PCs) via all-to-all BLASTp by DIAMOND (v2.1.10.164) with the default parameters of vConTACT (v2.0). The degree of similarity between the vOTUs was calculated based on the number of shared PCs. Then, pairs of closely related vOTUs with a similarity score of ≥ 1 were grouped into viral clusters (VCs), which were approximately at the genus level. The complete prokaryotic viral genomes (*n* = 5009) from the International Committee on Taxonomy of Viruses (ICTV) (VMR_MSL39.v2) [55] were included in the networks used as references. The networks were visualized by Cytoscape (v3.9.1) [56] using a prefuse force-directed model.

Sequence similarity between all proteins of vOTUs were searched by BLASTp using DIAMOND with parameters “--evalue 0.0001 --id 35 --query-cover 50 --subject-cover 50”. Then the weighted Gene Repertoire Relatedness (wGRR) was calculated as previously described [57]:

$$wGRR=\frac{\sum_{i}^{p} id(A_{i},B_{i})}{\min(A,B)}$$

where *A_i_* and *B_i_* are the pair *I* of homologous proteins present in *A* and *B*, respectively; *id (A_i_, B_i_)* is their sequence identity in the local alignment; min (*A, B*) is the number of proteins of the genome encoding the fewest proteins (*A* or *B*).

**Enrichment analysis**

The enrichment analyses on the distribution of viral host, derived environment, and protein function were performed as previously described [58]. Taking host distribution of SdPs as an example, the fold enrichment was calculated by dividing the proportion of hosts carrying SdPs in a specific taxonomy by the proportion of that host taxonomy within the bacterial population. Statistical significance of the enrichment was assessed using Fisher’s exact test, followed by Benjamini-Hochberg false discovery rate (FDR) correction for two-tailed *p* values using the Python packages scipy.stats (v.1.9.1) [59] and statsmodels (v.0.13.2) [60]. Their biomes were determined by extracting the biosample information of the host genomes from the NCBI database.

**Analysis of genomic features and growth characteristics**

The GC content of intergenic regions within prophages and host genomes was computed using the custom script “get_gc_and_narsc.py” [61]. Protein-coding density was determined by quantifying the number of predicted proteins per kilobase of genome sequence. Codon usage bias was analyzed through the calculation of relative synonymous codon usage (RSCU) frequencies utilizing the “uco” function in the R package seqinr [62]. Genome-wide maximal growth rates of prokaryotic organisms were predicted using gRodon (v2.0) [63], an R package that employs codon usage bias patterns for growth rate estimation. Ribosomal protein-encoding genes, which are characteristically highly expressed in prokaryotic systems, were identified through BLASTp analysis against the bacterial ribosomal protein database (br01610) in the Kyoto Encyclopedia of Genes and Genomes (KEGG) [64]. The alignment parameters were set with stringent thresholds: E-value < 1e-10, sequence identity > 30%, and coverage > 50%. Minimal doubling time (MDT) was subsequently calculated using the “predictGrowth” function in gRodon, incorporating both whole genome and ribosomal protein gene datasets as input parameters. Based on empirical observations that fast-growing marine copiotrophs typically exhibit maximum growth rates exceeding 1 d^-1^, and considering the practical accuracy limitations of MDT predictions beyond 5 h [63,65], we defined prokaryotes with predicted MDT < 5 hours as fast-growing microorganisms in our analysis. We predicted the optimal growth temperature (OGT), pH, and salinity of bacterial genomes using GenomeSPOT (v1.0.1) [66]. Additionally, the OGT was further validated using Tome (v1.0.0) [67].

**Calculation of prophage-host dissimilarity**

The nucleotide and amino acid dissimilarity metrics between prophage and prokaryotic hosts were quantified following established computational methodologies [68]. Specifically, the oligonucleotide frequency dissimilarities (*d_2_** dissimilarity, *d_2_* dissimilarity, *d_2_^S^* dissimilarity, Euclidean distance (*Eu*), *EuF* distance, *Hao*, Jensen-Shannon divergence (*JS*), Manhattan distance (*Ma*), and Chebyshev distance (*Ch*)) at *k* = 6 were employed to assess nucleotide-level divergence between viral and host genomes, implemented through VirHostMatcher [69] with default parameters. Additionally, amino acid dissimilarity was characterized by calculating the cosine distance between codon frequency vectors (D_c_(phage, host)) for prophage and host sequences [62].

**Evaluating PSOSP performance on genomes of varying quality**

To evaluate PSOSP’s performance on bacterial and prophage genomes of varying quality, we generated benchmark datasets using 1000 randomly selected bacterial genomes (containing 3599 prophages) from our analysis. Following a CheckM-like approach [70], we simulated 3000 bacterial genomes with 50−100% completeness and 0−20% contamination by fragmenting source genomes into 5−50 kb non-overlapping windows, randomly removing contigs until reaching target completeness levels, then introducing contamination by adding random fragments from other randomly selected genomes until contamination thresholds were met. For prophages, we simulated 10,797 genomes (CheckV-like methodology [3]) with 50−100% completeness and 0−50% contamination by selecting variable-length sub-fragments to meet completeness targets and adding variable-length flanking host sequences at both ends to achieve contamination thresholds.

**Statistical analysis**

All statistical analyses and visualization were performed using R v4.2.1 [71] and Python v3.12 [72], unless otherwise stated. We evaluated the performance of seven clustering methods (Mean Shift, K-means, Birch, Gaussian Mixture, Bayesian Gaussian Mixture, Agglomerative Clustering, and Spectral Clustering) from the Python scikit-learn package (v1.6.1) [1] on the 24 validated CSBs in *E. coli* K12. The two-sided unpaired Welch’s *t* test between groups in all violin plots and box plots were performed using the ggsignif (v0.6.4) [73] package in R. Simple linear regression was performed to evaluate associations between mock genome quality and PSOSP detection performance. Statistical significance of the linear relationship was determined by testing the slope coefficient (two-sided *t*-test) using ggplot2 (v3.5.1) [73] and ggpubr (v0.6.0) [74] package in R. Distribution similarity of genome sizes, completeness and phage-specific functions between SiPs and SdPs was evaluated using Kolmogorov-Smirnov tests with Python function “stats” from Scipy (v1.13.1) [59]. Genome similarity of prophages was visualized using clinker (v0.0.31) [75]. Genome map of prophages was visualized using online platform Chiplot (<https://www.chiplot.online/>).

**References**

1. Pedregosa, Fabian, Gael Varoquaux, Alexandre Gramfort, Vincent Michel, Bertrand Thirion, Olivier Grisel, Mathieu Blondel, et al. 2011. “Scikit-learn: Machine Learning in Python.” *Journal of Machine Learning Research* 12: 2825-2830.

2. Dieppa-Colón, Etan, Cody Martin, James C. Kosmopoulos, Karthik Anantharaman. 2025. “Prophage-DB: a comprehensive database to explore diversity, distribution, and ecology of prophages.” *Environmental Microbiome* 20: 5. <https://doi.org/10.1186/s40793-024-00659-1>

3. Nayfach, Stephen, Antonio Pedro Camargo, Frederik Schulz, Emiley Eloe-Fadrosh, Simon Roux, Nikos C. Kyrpides. 2021. “CheckV assesses the quality and completeness of metagenome-assembled viral genomes.” *Nature Biotechnology* 39: 578-585. <https://doi.org/10.1038/s41587-020-00774-7>

4. Erill, Ivan, Susana Campoy, Jordi Barbé. 2007. “Aeons of distress: an evolutionary perspective on the bacterial SOS response.” *FEMS Microbiology Reviews* 31: 637-656. <https://doi.org/10.1111/j.1574-6976.2007.00082.x>

5. Marin, Julie, Fabia U. Battistuzzi, Anais C. Brown, S. Blair Hedges. 2016. “The timetree of prokaryotes new insights into their evolution and speciation.” *Molecular Biology and Evolution* 34: 437-446. <https://doi.org/10.1093/molbev/msw245>

6. Mazón, G., J. M. Lucen, S. Campoy, A. R. Fernández de Henestrosa, P. Candau, J. Barbé. 2004. “LexA-binding sequences in Gram-positive and cyanobacteria are closely related.” *Molecular Genetics and Genomics* 271: 40-49. <https://doi.org/10.1007/s00438-003-0952-x>

7. Mazón, Gerard, Ivan Erill, Susana Campoy, Pilar Cortés, Evelyne Forano, Jordi Barbé. 2004. “Reconstruction of the evolutionary history of the LexA-binding sequence.” *Microbiology* 150: 3783-3795. <https://doi.org/10.1099/mic.0.27315-0>

8. Mahmoud, Aminu Abdullahi, Xiaoyu Wang, Xinyu Liao, Song Zhang, Tian Ding, Juhee Ahn. 2025. “Impact of prophages on gut microbiota and disease associations.” *Microbial Pathogenesis* 204: 107642. <https://doi.org/10.1016/j.micpath.2025.107642>

9. Wong, Joel W. H., Emily P. Balskus. 2025. “Small molecules as modulators of phage–bacteria interactions.” *Current Opinion in Chemical Biology* 84: 102566. <https://doi.org/10.1016/j.cbpa.2024.102566>

10. Helfrich, Stefan, Eugen Pfeifer, Christina Krämer, Christian Carsten Sachs, Wolfgang Wiechert, Dietrich Kohlheyer, Katharina Nöh, Julia Frunzke. 2015. “Live cell imaging of SOS and prophage dynamics in isogenic bacterial populations.” *Molecular Microbiology* 98: 636-650. <https://doi.org/10.1111/mmi.13147>

11. Rokney, Assaf, Oren Kobiler, Amnon Amir, Donald L. Court, Joel Stavans, Sankar Adhya, Amos B. Oppenheim. 2008. “Host responses influence on the induction of lambda prophage.” *Molecular Microbiology* 68: 29-36. <https://doi.org/10.1111/j.1365-2958.2008.06119.x>

12. Shkilnyj, Paul, Gerald B. Koudelka. 2007. “Effect of salt shock on stability of λ*^imm434^* lysogens.” *Journal of Bacteriology* 189: 3115-3123. <https://doi.org/10.1128/JB.01857-06>

13. Silpe, Justin E., Bonnie L. Bassler. 2019. “A host-produced quorum-sensing autoinducer controls a phage lysis-lysogeny decision.” *Cell* 176: 268-280. <https://doi.org/10.1016/j.cell.2018.10.059>

14. Yang, M.-K., Y.-C. Yang, C.-H. Hsu. 2002. “Characterization of *Xanthomonas axonopodis* pv. citri LexA: recognition of the LexA binding site.” *Molecular Genetics and Genomics* 268: 477-487. <https://doi.org/10.1007/s00438-002-0754-6>

15. Erill, Ivan, Marcos Escribano, Susana Campoy, Jordi Barbé. 2003. “In silico analysis reveals substantial variability in the gene contents of the gamma proteobacteria LexA-regulon.” *Bioinformatics* 19: 2225-2236. <https://doi.org/10.1093/bioinformatics/btg303>

16. Abella, Marc, Ivan Erill, Mónica Jara, Gerard Mazón, Susana Campoy, Jordi Barbé. 2004. “Widespread distribution of a *lexA*-regulated DNA damage-inducible multiple gene cassette in the Proteobacteria phylum.” *Molecular Microbiology* 54: 212-222. <https://doi.org/10.1111/j.1365-2958.2004.04260.x>

17. Silpe, Justin E., Olivia P. Duddy, Grace E. Johnson, Grace A. Beggs, Fatima A. Hussain, Kevin J. Forsberg, Bonnie L. Bassler. 2023. “Small protein modules dictate prophage fates during polylysogeny.” *Nature* 620: 625-633. <https://doi.org/10.1038/s41586-023-06376-y>

18. Zhang, Adrianna P. P., Ying Z. Pigli, Phoebe A. Rice. 2010. “Structure of the LexA-DNA complex and implications for SOS box measurement.” *Nature* 466: 883-886. <https://doi.org/10.1038/nature09200>

19. Lozada-Chavez, Irma, Sarath Chandra Janga, Julio Collado-Vides. 2006. “Bacterial regulatory networks are extremely flexible in evolution.” *Nucleic Acids Research* 34: 3434-3445. <https://doi.org/10.1093/nar/gkl423>

20. Yona, Avihu H., Eric J. Alm, Jeff Gore. 2018. “Random sequences rapidly evolve into de novo promoters.” *Nature Communications* 9: 1530. <https://doi.org/10.1038/s41467-018-04026-w>

21. Hildebrand, Falk, Axel Meyer, Adam Eyre-Walker. 2010. “Evidence of selection upon genomic GC-content in bacteria.” *PLoS Genetics* 6: e1001107. <https://doi.org/10.1371/journal.pgen.1001107>

22. Almpanis, Apostolos, Martin Swain, Derek Gatherer, Neil McEwan. 2018. “Correlation between bacterial G+C content, genome size and the G+C content of associated plasmids and bacteriophages.” *Microbial Genomics* 4: e000168. <https://doi.org/10.1099/mgen.0.000168>

23. Consortium, The UniProt. 2025. “UniProt: the universal protein knowledgebase in 2025.” *Nucleic Acids Research* 53: D609–D617. <https://doi.org/10.1093/nar/gkae1010>

24. Li, Shan, Minli Xu, Zhengchang Su. 2010. “Computational analysis of LexA regulons in Cyanobacteria.” *BMC Genomics* 11: 527. <https://doi.org/10.1186/1471-2164-11-527>

25. Leão, Pedro, Mary E. Little, Kathryn E. Appler, Daphne Sahaya, Emily Aguilar-Pine, Kathryn Currie, Ilya J. Finkelstein, Valerie De Anda, Brett J. Baker. 2024. “Asgard archaea defense systems and their roles in the origin of eukaryotic immunity.” *Nature Communications* 15: 6386. <https://doi.org/10.1038/s41467-024-50195-2>

26. Lewis, L.Kevin, Greg R. Harlow, Leslie A. Gregg-Jolly, David W. Mount. 1994. “Identification of high affinity binding sites for LexA which define new DNA damage-inducible genes in *Escherichia coli*.” *Journal of Molecular Biology* 241: 507-523. <https://doi.org/10.1006/jmbi.1994.1528>

27. Bailey, Timothy L., James Johnson, Charles E. Grant, William S. Noble. 2015. “The MEME Suite.” *Nucleic Acids Research* 43: W39-49. <https://doi.org/10.1093/nar/gkv416>

28. Grant, Charles E., Timothy L. Bailey, William Stafford Noble. 2011. “FIMO: scanning for occurrences of a given motif.” *Bioinformatics* 27: 1017-1018. <https://doi.org/10.1093/bioinformatics/btr064>

29. Dougherty, Peter Erdmann, Tue Kjærgaard Nielsen, Leise Riber, Helen Helgå Lading, Laura Milena Forero-Junco, Witold Kot, Jos M. Raaijmakers, Lars Hestbjerg Hansen. 2023. “Widespread and largely unknown prophage activity, diversity, and function in two genera of wheat phyllosphere bacteria.” *The ISME Journal* 17: 2415-2425. <https://doi.org/10.1038/s41396-023-01547-1>

30. Chen, Shifu, Yanqing Zhou, Yaru Chen, Jia Gu. 2018. “Fastp: an ultra-fast all-in-one FASTQ preprocessor.” *Bioinformatics* 34: i884-i890. <https://doi.org/10.1093/bioinformatics/bty560>

31. Li, Heng. 2013. “Aligning sequence reads, clone sequences and assembly contigs with BWA-MEM.” *arXiv*: 1303.3997v1302. <https://doi.org/10.48550/arXiv.1303.3997>

32. Thorvaldsdóttir, Helga, James T Robinson, Jill P Mesirov. 2013. “Integrative genomics viewer (IGV): high-performance genomics data visualization and exploration.” *Briefings in Bioinformatics* 14: 178-192. <https://doi.org/10.1093/bib/bbs017>

33. Haft, Daniel H., Azat Badretdin, George Coulouris, Michael DiCuccio, A. Scott Durkin, Eric Jovenitti, Wenjun Li, et al. 2024. “RefSeq and the prokaryotic genome annotation pipeline in the age of metagenomes.” *Nucleic Acids Research* 52: D762–D769. <https://doi.org/10.1093/nar/gkad988>

34. O'Leary, Nuala A., Mathew W. Wright, J. Rodney Brister, Stacy Ciufo, Diana Haddad, Rich McVeigh, Bhanu Rajput, et al. 2016. “Reference sequence (RefSeq) database at NCBI: current status, taxonomic expansion, and functional annotation.” *Nucleic Acids Research* 44: D733-745. <https://doi.org/10.1093/nar/gkv1189>

35. Camargo, Antonio Pedro, Simon Roux, Frederik Schulz, Michal Babinski, Yan Xu, Bin Hu, Patrick S. G. Chain, Stephen Nayfach, Nikos C. Kyrpides. 2024. “Identification of mobile genetic elements with geNomad.” *Nature Biotechnology* 42: 1303-1312. <https://doi.org/10.1038/s41587-023-01953-y>

36. Zhang, Mujie, Yali Hao, Yi Yi, Shunzhang Liu, Qingyang Sun, Xiaoli Tan, Shan Tang, Xiang Xiao, Huahua Jian. 2023. “Unexplored diversity and ecological functions of transposable phages.” *The ISME Journal* 17: 1015-1028. <https://doi.org/10.1038/s41396-023-01414-z>

37. Camacho, Christiam, George Coulouris, Vahram Avagyan, Ning Ma, Jason Papadopoulos, Kevin Bealer, Thomas L Madden. 2009. “BLAST+: architecture and applications.” *BMC Bioinformatics* 10: 421. <https://doi.org/10.1186/1471-2105-10-421>

38. Hyatt, Doug, Gwo-Liang Chen, Philip F LoCascio, Miriam L Land, Frank W Larimer, Loren J Hauser. 2010. “Prodigal: prokaryotic gene recognition and translation initiation site identification.” *BMC Bioinformatics* 11: 119. <https://doi.org/10.1186/1471-2105-11-119>

39. Buchfink, Benjamin, Chao Xie, Daniel H Huson. 2015. “Fast and sensitive protein alignment using DIAMOND.” *Nature Methods* 12: 59-60. <https://doi.org/10.1038/nmeth.3176>

40. Camargo, Antonio Pedro, Stephen Nayfach, I-Min A. Chen, Krishnaveni Palaniappan, Anna Ratner, Ken Chu, Stephan J. Ritter, et al. 2023. “IMG/VR v4: an expanded database of uncultivated virus genomes within a framework of extensive functional, taxonomic, and ecological metadata.” *Nucleic Acids Research* 51: D733-D743. <https://doi.org/10.1093/nar/gkac1037>

41. Pinto, Yishay, Meenakshi Chakraborty, Navami Jain, Ami S. Bhatt. 2024. “Phage-inclusive profiling of human gut microbiomes with Phanta.” *Nature Biotechnology* 42: 651–662. <https://doi.org/10.1038/s41587-023-01799-4>

42. Liao, Hu, Jian Li, Yan-Zi Wang, Hu Li, Xin-Li An, Tao Wang, Rui-Ying Chang, Yong-Guan Zhu, Jian-Qiang Su. 2025. “Evolutionary diversification and succession of soil huge phages in glacier foreland.” *Microbiome* 13: 18. <https://doi.org/10.1186/s40168-024-02017-2>

43. Roux, Simon, Mart Krupovic, Rebecca A. Daly, Adair L. Borges, Stephen Nayfach, Frederik Schulz, Allison Sharrar, et al. 2019. “Cryptic inoviruses revealed as pervasive in bacteria and archaea across Earth's biomes.” *Nature Microbiology* 4: 1895-1906. <https://doi.org/10.1038/s41564-019-0510-x>

44. Reiter, Wolf-Dieter, Peter Palm. 1990. “Identification and characterization of a defective SSV1 genome integrated into a tRNA gene in the archaebacterium *Sulfolobus* sp. B12.” *Molecular and General Genetics* 221: 65-71. <https://doi.org/10.1007/BF00280369>

45. Ooka, Tadasuke, Yoshitoshi Ogura, Md Asadulghani, Makoto Ohnishi, Keisuke Nakayama, Jun Terajima, Haruo Watanabe, Tetsuya Hayashi. 2009. “Inference of the impact of insertion sequence (IS) elements on bacterial genome diversification through analysis of small-size structural polymorphisms in *Escherichia coli* O157 genomes.” *Genome Research* 19: 1809–1816. <https://doi.org/10.1101/gr.089615.108>

46. Zhou, Wenyuan, Yajie Li, Xuechao Xu, Shengqi Rao, Hua Wen, Yeiling Han, Aiping Deng, Zhenwen Zhang, Zhenquan Yang, Guoqiang Zhu. 2023. “Whole-genome analysis showed the promotion of genetic diversity and coevolution in *Staphylococcus aureus* lytic bacteriophages and their hosts mediated by prophages *via* worldwide recombination events.” *Frontiers in Microbiology* 14: 1088125. <https://doi.org/10.3389/fmicb.2023.1088125>

47. Bobay, Louis-Marie, Marie Touchon, Eduardo P. C. Rocha. 2014. “Pervasive domestication of defective prophages by bacteria.” *Proceedings of the National Academy of Sciences* 111: 12127-12132. <https://doi.org/10.1073/pnas.1405336111>

48. Chaumeil, Pierre-Alain, Aaron J. Mussig, Philip Hugenholtz, Donovan H. Parks. 2022. “GTDB-Tk v2: memory friendly classification with the genome taxonomy database.” *Bioinformatics* 38: 5315-5316. <https://doi.org/10.1093/bioinformatics/btac672>

49. Price, Morgan N., Paramvir S. Dehal, Adam P. Arkin. 2010. “FastTree 2 – approximately maximum-likelihood treesfor large alignments.” *PloS One* 5: e9490. <https://doi.org/10.1371/journal.pone.0009490>

50. Xie, Jianmin, Yuerong Chen, Guanjing Cai, Runlin Cai, Zhong Hu, Hui Wang. 2023. “Tree Visualization By One Table (tvBOT): a web application for visualizing, modifying and annotating phylogenetic trees.” *Nucleic Acids Research* 51: W587-W592. <https://doi.org/10.1093/nar/gkad359>

51. Roux, Simon, Evelien M Adriaenssens, Bas E Dutilh, Eugene V Koonin, Andrew M Kropinski, Mart Krupovic, Jens H Kuhn, et al. 2019. “Minimum information about an uncultivated virus genome (MIUViG).” *Nature Biotechnology* 37: 29-37. <https://doi.org/10.1038/nbt.4306>

52. Nayfach, Stephen, David Páez-Espino, Lee Call, Soo Jen Low, Hila Sberro, Natalia N. Ivanova, Amy D. Proal, et al. 2021. “Metagenomic compendium of 189,680 DNA viruses from the human gut microbiome.” *Nature Microbiology* 6: 960-970. <https://doi.org/10.1038/s41564-021-00928-6>

53. Enright, A. J., S. Van Dongen, C. A. Ouzounis. 2002. “An efficient algorithm for large-scale detection of protein families.” *Nucleic Acid Research* 30: 1575-1584. <https://doi.org/10.1093/nar/30.7.1575>

54. Jang, Ho Bin, Benjamin Bolduc, Olivier Zablocki, Jens H. Kuhn, Simon Roux, Evelien M. Adriaenssens, J. Rodney Brister, et al. 2019. “Taxonomic assignment of uncultivated prokaryotic virus genomes is enabled by gene-sharing networks.” *Nature Biotechnology* 37: 632-639. <https://doi.org/10.1038/s41587-019-0100-8>

55. Lefkowitz, Elliot J., Donald M. Dempsey, Robert Curtis Hendrickson, Richard J. Orton, Stuart G. Siddell, Donald B. Smith. 2018. “Virus taxonomy: the database of the International Committee on Taxonomy of Viruses (ICTV).” *Nucleic Acids Research* 46: D708-D717. <https://doi.org/10.1093/nar/gkx932>

56. Shannon, Paul, Andrew Markiel, Owen Ozier, Nitin S. Baliga, Jonathan T. Wang, Daniel Ramage, Nada Amin, Benno Schwikowski, Trey Ideker. 2003. “Cytoscape a software environment for integrated models of biomolecular interaction networks.” *Genome Research* 13: 2498-2504. <https://doi.org/10.1101/gr.1239303>

57. Sousa, Jorge A. Moura de, Alfred Fillol-Salom, Jose R. Penades, Eduardo P.C. Rocha. 2023. “Identification and characterization of thousands of bacteriophage satellites across bacteria.” *Nucleic Acids Research* 51: 2759-2777. <https://doi.org/10.1093/nar/gkad123>

58. Hsu, Tiffany Y., Etienne Nzabarushimana, Dennis Wong, Chengwei Luo, Robert G. Beiko, Morgan Langille, Curtis Huttenhower, Long H. Nguyen, Eric A. Franzosa. 2025. “Profiling lateral gene transfer events in the human microbiome using WAAFLE.” *Nature Microbiology* 10: 94-111. <https://doi.org/10.1038/s41564-024-01881-w>

59. Virtanen, Pauli, Ralf Gommers, Travis E. Oliphant, Matt Haberland, Tyler Reddy, David Cournapeau, Evgeni Burovski, et al. 2020. “SciPy 1.0: fundamental algorithms for scientific computing in Python.” *Nature Methods* 17: 261-272. <https://doi.org/10.1038/s41592-019-0686-2>

60. Seabold, Skipper, Josef Perktold. 2010. “Statsmodels: econometric and statistical modeling with Python.” *9th Python in Science Conference*, Austin, pp. 57-61.

61. Getz, Eric W., Saima Sultana Tithi, Liqing Zhang, Frank O. Aylward. 2018. “Parallel evolution of genome streamlining and cellular bioenergetics across the marine radiation of a bacterial phylum.” *mBio* 9: e01089-01018. <https://doi.org/10.1128/mbio.01089-18>

62. Howard-Varona, Cristina, Morgan M. Lindback, G. Eric Bastien, Natalie Solonenko, Ahmed A. Zayed, HoBin Jang, Bill Andreopoulos, et al. 2020. “Phage-specific metabolic reprogramming of virocells.” *The ISME Journal* 14: 881-895. <https://doi.org/10.1038/s41396-019-0580-z>

63. Weissman, Jake L., Shengwei Hou, Jed A. Fuhrman. 2021. “Estimating maximal microbial growth rates from cultures, metagenomes, and single cells via codon usage patterns.” *Proceedings of the National Academy of Sciences* 118: e2016810118. <https://doi.org/10.1073/pnas.2016810118>

64. Kanehisa, Minoru, Miho Furumichi, Yoko Sato, Mari Ishiguro-Watanabe, Mao Tanabe. 2021. “KEGG: integrating viruses and cellular organisms.” *Nucleic Acids Research* 49: D545-D551. <https://doi.org/10.1093/nar/gkaa970>

65. Kirchman, David L. 2016. “Growth rates of microbes in the oceans.” *Annual Reviews Marine Science* 8: 285-309. <https://doi.org/10.1146/annurev-marine-122414-033938>

66. Li, Gang, Kersten S. Rabe, Jens Nielsen, Martin K. M. Engqvist. 2019. “Machine learning applied to predicting microorganism growth temperatures and enzyme catalytic optima.” *ACS Synthetic Biology* 8: 1411-1420. <https://doi.org/10.1021/acssynbio.9b00099>

67. Barnum, Tyler P., Alexander Crits-Christoph, Michael Molla, Paul Carini, Henry H. Lee, Nili Ostrov. 2024. “Predicting microbial growth conditions from amino acid composition.” *BioRxiv*. <https://doi.org/10.1101/2024.03.22.586313>

68. Jian, Huahua, Yi Yi, Jiahua Wang, Yali Hao, Mujie Zhang, Siyuan Wang, Canxing Meng, et al. 2021. “Diversity and distribution of viruses inhabiting the deepest ocean on Earth.” *The ISME Journal* 15: 3094-3110. <https://doi.org/10.1038/s41396-021-00994-y>

69. Ahlgren, Nathan A., Jie Ren, Yang Young Lu, Jed A. Fuhrman, Fengzhu Sun. 2017. “Alignment-free *d_2_** oligonucleotide frequency dissimilarity measure improves prediction of hosts from metagenomically-derived viral sequences.” *Nucleic Acids Research* 45: 39-53. <https://doi.org/10.1093/nar/gkw1002>

70. Parks, Donovan H., Michael Imelfort, Connor T. Skennerton, Philip Hugenholtz, Gene W. Tyson. 2015. “CheckM: assessing the quality of microbial genomes recovered from isolates, single cells, and metagenomes.” *Genome Research* 25: 1043-1055. <https://doi.org/10.1101/gr.186072.114>

71. Team, R Core. 2013. “R: A language and environment for statistical computing.” R Foundation for Statistical Computing, Vienna, Austria.

72. Oliphant, Travis E. 2007. “Python for scientific computing.” *Computing in Science & Engineering* 9: 10-20. <https://doi.org/10.1109/MCSE.2007.58>

73. Ahlmann-Eltze, Constantin, Indrajeet Patil. 2021. “Ggsignif: R package for displaying significance brackets for ‘ggplot2’.” *PsyArXiv*. <https://doi.org/doi:10.31234/osf.io/7awm6>

74. Kassambara, Alboukadel. 2025. “ggpubr: 'ggplot2' based publication ready plots.” *R package version* (2018): 2. <https://rpkgs.datanovia.com/ggpubr/>

75. Gilchrist, Cameron L. M., Yit-Heng Chooi. 2021. “Clinker & clustermap.js: Automatic generation of gene cluster comparison figures.” *Bioinformatics* 37: 2473-2475. <https://doi.org/10.1093/bioinformatics/btab007>

**Figure S1 Classification of *HI* for all PSBs in *E. coli* K12 using different clustering methods.** Experimentally validated PSBs capable and incapable of LexA binding are represented by blue and orange squares, respectively. Red dashed lines indicate the upper threshold of the low-*HI* category (*HI_C1_*) and the lower threshold of the high-*HI* category (*HI_C2_*).

**Figure S2 Validation of the SOS-dependency of prophage SW1 in *S. piezotolerans* WP3. (A)** Three PSBs identified in the SW1 genome. The positions of PSBs in the SW1 genome are marked by red bars, with their corresponding DNA sequences displayed below (conserved sites are highlighted in red). **(B)** Distribution of *HI* values for all PSBs in *S. piezotolerans* WP3. Experimentally validated PSBs (located on prophage SW1) that can and cannot bind LexA are represented by blue and orange squares, respectively. (C) Electrophoretic mobility shift assay (EMSA) assessing the binding of LexA to the three PSBs. White and black triangles indicate the bound and unbound probes, respectively. Binding specificity was assessed using non-specific probes (NSP) and specific probes (SP) in the competitive EMSA. (D) Three sets of mutant sequences (M1-M3) designed for the three PSBs, along with their *HI* values. (E) EMSA analysis of LexA binding to the nine mutant PSB sequences. (F) qPCR analysis of the effects of UV irradiation, mitomycin C (MMC) treatment, and *lexA* gene mutation on the transcription of SOS pathway key genes (*recA*, *lexA*) and SW1 genes. Transcription fold changes were normalized to the untreated group or ***S.* *piezotolerans* WP3** wild-type strain. The dashed line indicates a fold change of 1. Data are presented as mean ± s.d. based on three independent biological replicates. Statistical significance was determined by Welch’s *t*-test.

**Figure S3 Distribution of PSBs and *HI* values for experimentally validated eight SdPs and two SiPs.** (A) PSBs identified in the genomes of three prophages (P22, Fels1, and Fels2) in *Salmonella typhimurium* LT2 (left panel) and their corresponding *HI* distribution (right panel). (B) PSB identified in the genome of prophage ФЕСО1 in *Escherichia coli* HS (left panel) and its *HI* distribution (right panel). (C) PSB identified in the genome of prophage ST2-8624 in *Escherichia coli* O157:H7 (left panel) and its *HI* distribution (right panel). (D) PSB identified in the genome of prophage VALG_phi6 in *Vibrio alginolyticus* K01M1 (left panel) and its *HI* distribution (right panel). (E) PSB identified in the genome of prophage B3 in *Pseudomonas aeruginosa* PAO1 (left panel) and its *HI* distribution (right panel). (F) PSB identified in the genome of prophage vB_SspM_BZS1 and vB_SspS_OS31 in *Serratia* sp. OS31 (left panel) and its *HI* distribution (right panel). (G) PSB identified in the genome of prophage yong1 in *Hafnia paralvei* LY-23 (left panel) and its *HI* distribution (right panel). PSB positions within the prophages are indicated by red bars, with their DNA sequences displayed below the corresponding positions (conserved sites are highlighted in red).

**Figure S4 Validation of the SOS-dependency of the three prophages in *S. psychrophila* WP2.** (A) PSBs identified in the genomes of three prophages (SP1, SP2, and SP3). The positions of PSBs in the prophages are indicated by red bars, with their DNA sequences displayed below the corresponding positions (conserved sites are highlighted in red). (B) Distribution of *HI* values for all PSBs in *S. psychrophila* WP2. The PSBs located on three prophages (SP1, SP2, and SP3) are represented by blue and orange squares according to their respective *HI* values. (C) VIP-Seq analysis of WP2 genome sequence depth in the mitomycin C (MMC) treated group (yellow) and untreated control group (brown). The experiment included three independent biological replicates for the MMC-treated group and two independent biological replicates for the untreated control group. (D) VIP-Seq analysis of phage titres for SP1, SP2, and SP3. Data are presented as mean ± s.d. based on three independent biological replicates for the MMC-treated group and two for the untreated control group. Statistical significance was determined by two-sided unpaired Welch’s *t*-test.

**Figure S5 Genome completeness and length distribution of prophages from different sources.** (A and B) Density distribution (A) and pairwise comparison (B) of genome completeness among three prophage sources. (C and D) Density distribution (C) and pairwise comparison (D) of genome lengths among the same three groups. Each box represents the interquartile range (IQR), in which the middle line represents the median. The whiskers extend to 1.5 × IQR. The significances were analyzed by the two-sided unpaired Welch’s *t*-test, and the *p*-value is shown above boxes. For clarity, only prophages with genome lengths less than 100 kb are shown in the length analysis. Qualified refers to prophages that passed the quality control criteria in this study; Unqualified refers to those that did not; Prophage-DB represents prophages from a previously published database [2].

**Figure S6 Comparison of *HI* values across bacterial genomes of different taxonomic levels.** (A) Distribution of *HI* values in the top 10 most abundant bacterial species. *HI_C1_* indicates the upper threshold of the low-*HI* category, and *HI_C2_* indicates the lower threshold of the high-*HI* category. In the dumbbell plots, blue and white dots represent the average *HI_C1_* and *HI_C2_* values, respectively, across all genomes within each genus. (B) Distribution of *HI* values in the top 10 most abundant bacterial genera. (C−E) Statistical comparison of *HI* values among bacterial genomes at the species (C), genus (D), and family (E) levels. In each heatmap, the lower triangle represents significance levels for *HI_C1_*, and the upper triangle for *HI_C2_*. Significance was determined by two-sided unpaired Welch’s *t*-test. Asterisks denote *p* values: no asterisk indicates *p* ≥ 0.05, **p* < 0.05, ***p* < 0.01, ****p* < 0.001.

**Figure S7 Distribution of *HI* clusters in bacteria.** (A) Schematic diagram illustrating the construction process of the provirus-host dataset. The workflow comprises two main steps: prophage prediction and precise delineation of prophage boundaries. (B) Distribution of bacteria containing canonical SOS boxes (CSBs) at the class level. (C) Distribution of bacteria containing different number of *HI* clusters. (D) Distribution of *HI* in representative bacterial strains with various numbers of *HI* clusters (cluster 1−6). (E) Comparative analysis of *HI* distributions among the three types of prophages (SiPs, SdPs, and SuPs). In each boxplot, the box represents the interquartile range (IQR), the black central line indicates the median, and the whiskers extend to 1.5 × IQR. Statistical significance of differences was assessed using a two-sided unpaired Welch’s *t*-test.

**Figure S8 Genera retained and filtered out in the analysis of bacterial genomes from the RefSeq database (Release 226) by PSOSP.** (A) Genera retained in the analysis, showing only the top 20 genera with the highest number of retained genomes. (B) Genera filtered out from the analysis, showing only the top 20 genera with the highest number of filtered genomes. The total number of bacterial genomes in each genus is indicated above each bar.

**Figure S9 Performance evaluation of PSOSP on host and phage genomes of varying quality.** (A) Effect of host genome quality on PSOSP prediction performance. Upper panels: genome completeness vs. success rate and precision. Lower panels: genome contamination vs. success rate and precision. Success rate is defined as the proportion of prophages for which PSOSP successfully made predictions. (B) Effect of prophage genome quality on PSOSP prediction performance. Layout is the same as in(A). Statistical significance of the linear relationships was assessed using two-sided *t*-tests. *R^2^* and *p*-values from linear regression analyses are shown in each subplot.

**Figure S10 Consistency of regulatory patterns among multiple prophages in polylysogenic strains.** (A) Distribution of polylysogenic and monolysoegenic strains. (B) Distribution of prophage counts in polylysogenic strains. (C) Regulatory consistency among prophages in polylysogenic strains. For clarity, only genera with more than 15 genomes are shown. The regulatory consistency score was calculated as (*n_1_* × 0.5 + *n_2_* × 1) / *N*, where *n_1_* represents the number of SuPs, *n_2_* represents the number of SiPs, and *N* represents the total number of prophages in the strain. A higher score indicates a greater tendency for prophages in the strain to be SOS-independent.

**Figure S11 Viral similarity analysis based on wGRR.** (A) Shared network of SiPs and SdPs constructed based on wGRR. Nodes and connecting edges represent vOTUs and their wGRR scores, respectively. Nodes are depicted in the color representing vOTUs of SiPs (orange) and SdPs (blue). (B) Distribution of wGRR scores within or between SiPs and SdPs. Statistical significance tests were performed using two-sided unpaired Welch’s *t*-test, with *p*-values displayed above each plot. The solid and dashed black lines in the violin plots represents the median and the quartiles, respectively. Black triangle denotes the mean.

**Figure S12 Representative case of mutual conversion between SiP and SdP.** The positions of PSBs in the prophages are indicated by red bars, with their DNA sequences displayed below the corresponding positions (mutated nucleotide positions are highlighted in red). Grey/black bands connect homologous proteins, with shades of grey representing the percentage of amino-acid sequence identity. The fill colors of the arrows indicate different functional categories of the proteins.

**Figure S13 Distribution of SiPs, SuPs, and SdPs across different environments.** (A) Overall distribution of SiPs, SuPs, and SdPs in various environments. (B and C) Distribution within the top 2 sub-environments: human (B) and animal (C). The total number of prophages in each environment is indicated to the right of each bar.

**Figure S14 Comparison of genome size, host distribution, environmental distribution, and protein function categories between SdPs and SiPs.** (A and B) Comparison of genome size distribution (A) and completeness (B) of SdPs and SiPs. (C) Host distribution of SdPs and SiPs. For clarity, only the top ten abundant bacterial genus are shown, with the five most abundant species displayed for each genus. (D) Environmental distribution of SdPs and SiPs. (E) Protein function distribution of SdPs and SiPs. For clarity, only the five most abundant proteins in each functional category are shown. Statistical significance of the enrichment was assessed using Fisher’s exact test, followed by Benjamini-Hochberg false discovery rate (FDR) correction for two-tailed *p-*values. “*” and “**” indicate *p*-adjusted < 0.05 and < 0.01, respectively. (F) Presence of phage-specific functions in SdPs and SiPs.

**Figure S15 Comparative analysis of genomic and growth characteristics between SiPs hosts and SdPs hosts.** (A) Comparison of genomic features between SiPs and SdPs hosts. (B) Proportion of fast-growing strains (MDT < 5 h) among SiPs and SdPs hosts. (C) Predicted optimal growth temperature, pH, and salinity of SiPs and SdPs hosts. All statistical significance tests were performed using a two-sided unpaired Welch’s *t*-test, with *p*-values displayed above each plot. The solid and dashed black lines in the violin plots represents the median and the quartiles, respectively. The black triangles denote the means.

**Figure S16 Comparative analysis of nucleotide frequency divergence between SiPs and SdPs and their respective hosts.** The nucleotide frequency divergence between phages and hosts was calculated using VirHostMatcher [69], including *d_2_*, *d_2_^S^* dissimilarity, Euclidean distance (*Eu*), *EuF* distance, *Hao*, Jensen-Shannon divergence (*JS*), Manhattan distance (*Ma*), Chebyshev distance (*Ch*). All statistical significance tests were performed using a two-sided unpaired Welch’s *t*-test, with *p*-values displayed above each plot. The solid and dashed black lines in the violin plots represents the median and the quartiles, respectively. The black triangles denotes the means.
